# Liposarcoma of the Spermatic Cord Mimicking an Inguinal Hernia: A Case Report and Literature Review

**DOI:** 10.7759/cureus.28269

**Published:** 2022-08-22

**Authors:** Konstantinos Pikramenos, Stamatios Katsimperis, Maria Zachou, Maria Giannakakou, Maria Mitsogianni, Iraklis Mitsogiannis

**Affiliations:** 1 2nd Department of Urology, Sismanoglio General Hospital, National and Kapodistrian University of Athens, Athens, GRC; 2 Gastroenterology, Sismanoglio General Hospital, National and Kapodistrian University of Athens, Athens, GRC; 3 2nd Department of Internal Medicine-Propaedeutic, Oncology Section, Attikon University Hospital, Chaidari, GRC; 4 4th Department of Medical Oncology, Hygeia Hospital, Marousi, GRC

**Keywords:** sclerosing liposarcoma, myxoid liposarcoma, para-testicular tumor, orchidectomy, liposarcoma

## Abstract

Liposarcomas of the spermatic cord are extremely rare, with less than 200 cases in the literature. We present a case of sclerosing mixed with myxoid liposarcoma of the left spermatic cord in a 55-year-old male patient, mimicking an inguinal hernia on pre-operative ultrasound. The patient underwent orchidectomy and is currently on follow-up surveillance with no signs of recurrence.

## Introduction

Less than 200 cases of paratesticular liposarcomas have been reported so far in the literature [1-3]. Most paratesticular liposarcomas are primary; nonetheless, metastatic tumors originating from the thigh or the fatty tissue surrounding the testicle have also been described [4]. According to the WHO classification of soft tissue tumors, liposarcomas are classified into five categories: 1) well differentiated, including the adipocytic, inflammatory, and sclerosing subtypes, 2) dedifferentiated, 3) myxoid, 4) round cell, and 5) pleiomorphic. Round cell and pleiomorphic types have the worst prognosis, as they are associated with a higher rate of recurrence and hematogenous metastasis to the lung and bones. In contrast, differentiated and myxoid types are associated with locoregional recurrence [5-8]. We present a case of sclerosing mixed with myxoid liposarcoma of the left spermatic cord.

## Case presentation

**Figure 1:**
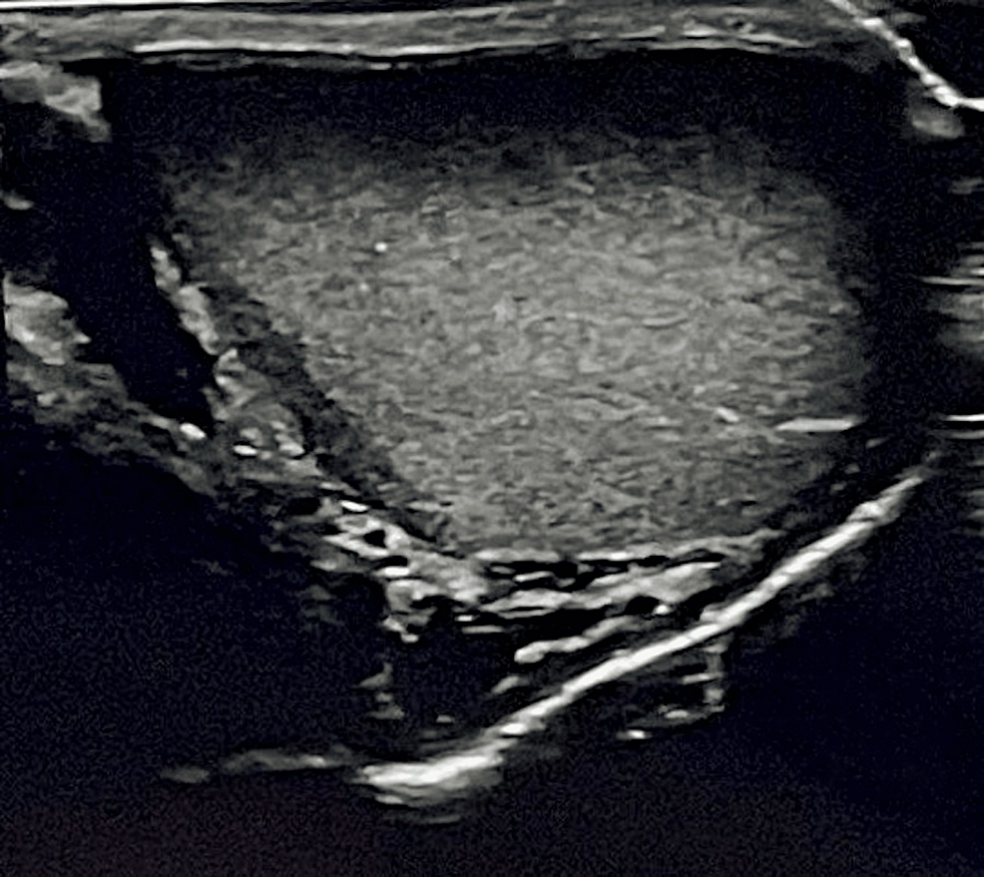
Ultrasonographic image of the left testicle.

**Figure 2:**
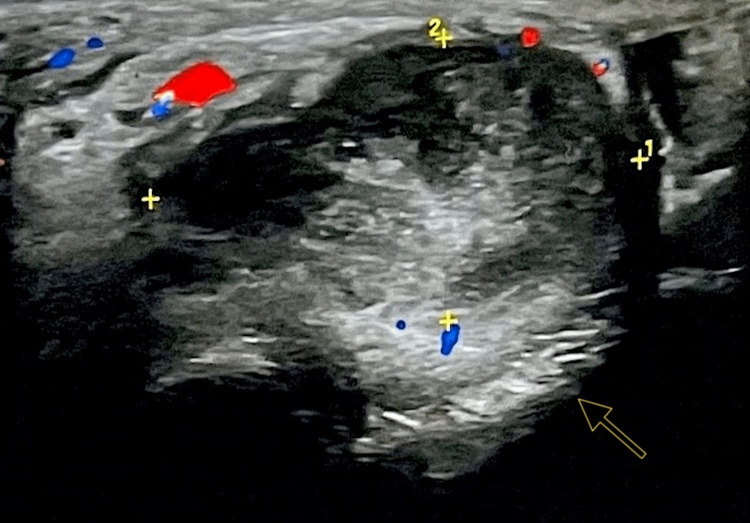
Ultrasonographic image of the left paratesticular lesion. Arrow indicating the paratesticular lesion, measuring (1) L 3,21 x (2) H 2,27. Doppler ultrasound measurements indicating blood supply of the lesion: - Red: Arteries - Blue: Veins

A 55-year-old male patient with a history of irritable bowel syndrome and generalized anxiety disorder was referred to the Urology clinic by his treating physician after he had noticed a “lump” in the area above his left testicle. On clinical examination, the “lump” was easily felt as a hard and painless nodule, highly suspicious of malignancy. USG revealed a normal left testicle (Figure 1) and a soft tissue nodule, measuring 3.88 cm in diameter, located over the upper pole of the testis, identified as an inguinal hernia (Figure 2).

**Figure 3:**
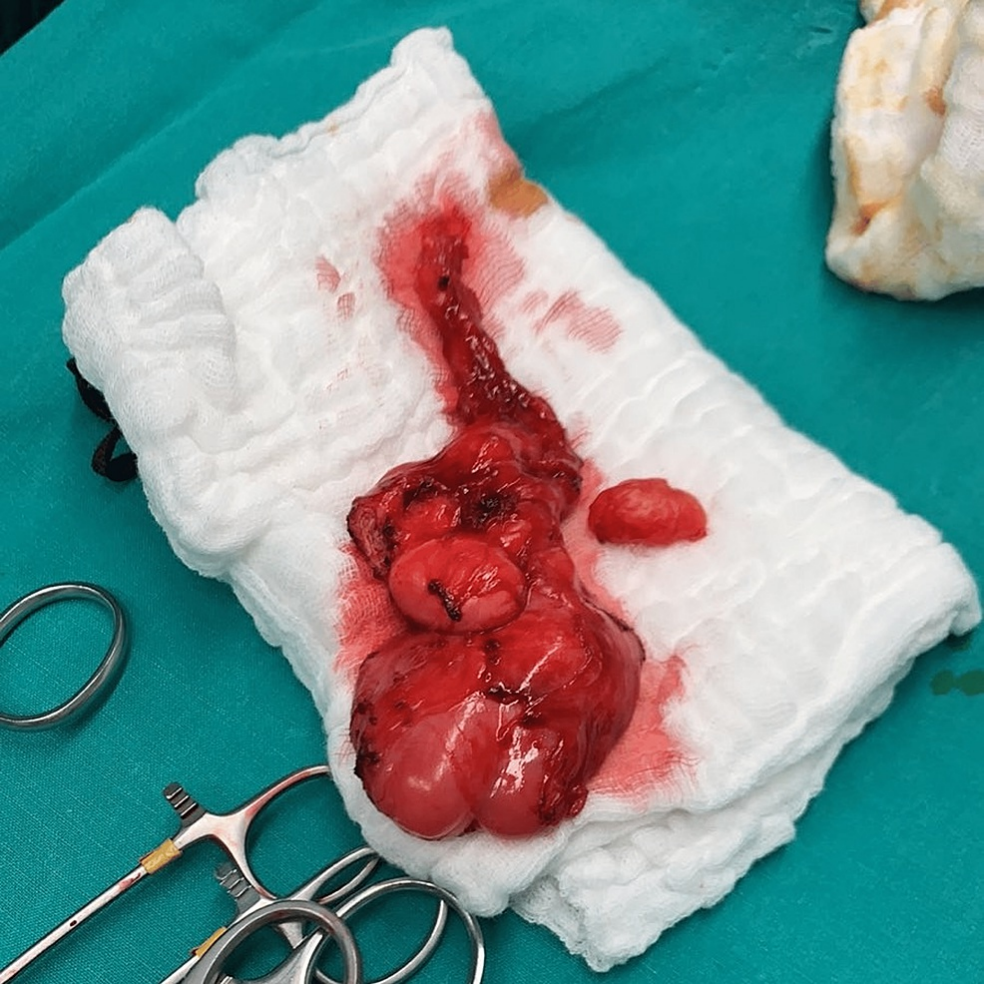
Left testicle and paratesticular lesion specimen.

CT of the chest and abdomen did not detect any signs of metastatic disease. Following discussion with the patient, who was informed about the high possibility of the tumor to be malignant and consented to the proposed treatment, a left orchidectomy, along with excision of the left spermatic cord up to the level of the internal inguinal ring, was carried out through an inguinal incision (Figure 3).

A scrotal drain was inserted and left for 18 hours, and the patient was discharged the next day with no complications. Histology revealed a mixed variety of well-differentiated sclerosing liposarcoma (grade I - Fédération Nationale des Centres de Lutte Contre le Cancer [FNCLCC] system), with myxoid liposarcoma (grade II - FNCLCC system) of the spermatic cord, with negative surgical margins. An oncology consultation was requested, and after a thorough discussion with the patient, it was decided not to undergo any adjuvant treatment and continue with active surveillance. The patient was followed up with regular clinic visits, and CT scans every three months, with no sign of local recurrence or metastatic disease at the 12th postoperative month.

## Discussion

Clinical diagnosis of the tumors of the spermatic cord can be difficult. These tumors usually present as unilateral, hard, slow-growing masses of the inguinal canal or the scrotum, varying sizes between 1.5 and 30 cm [9,10]. Clinical manifestation is nonspecific, with the patients complaining of a sensation of compression and scrotal heaviness due to the growing local mass. These tumors may sometimes be misdiagnosed as hydroceles or inguinal hernias. 

On ultrasound, liposarcomas appear as hyperechoic and heterogeneous solid formations. There are no pathognomonic sonographic features to differentiate benign from malignant lesions, and additional imaging, usually with CT, is often required [11,12]. Nonetheless, a definite diagnosis is made following surgical exploration. 

Radical orchidectomy with en-block resection of the tumor, as in our case, is the treatment of choice [13]. Wide local excision is recommended for paratesticular tumors to prevent local recurrence and tumor spread [14]. Peralta JP et al. [15] presented a case of a spermatic cord liposarcoma where organ-sparing surgery was performed. One and a half years postoperatively, no signs of recurrence were documented. This infrequent approach may be feasible in selected cases where risk factors for recurrence, such as tumor grade, size, depth of invasion, and, most importantly, surgical margin status, are not ominous. However, they are generally not recommended as a standard method of treatment.

Because of the high propensity of local recurrence after surgery [9], radiotherapy may be used as an adjunct form of treatment. However, its role remains to be determined. The presence of positive surgical margins, unsuccessful wide excision due to extended tumor bulk, high-grade tumors, and locoregional recurrences are commonly accepted as indications for radiotherapy [16]. Thus far, neoadjuvant radiotherapy is not well established as a standard form of care. In a recent study, Chowdhry VK et al. [17] underlined the negative effects of preoperative radiotherapy in terms of wound healing, opposing its use. Finally, the role of both neoadjuvant and adjuvant chemotherapy in the treatment of liposarcomas has to be further investigated in well-documented studies.

## Conclusions

Liposarcomas of the spermatic cord are rare and can be easily misdiagnosed for hernias or another groin pathology. Diagnosis is usually established following radical orchidectomy, which is the standard form of treatment; nonetheless, recurrences are common. The role of both radiotherapy and chemotherapy remains to be determined. Patients with liposarcomas of the spermatic cord require meticulous medical attention and close follow-up for the prevention of tumor relapse.
